# Chemical Control of Ichthyotoxic Algal Blooms in Aquaculture: Assessing Algicide Impacts on Cellular Motility and Bloom Suppression

**DOI:** 10.3390/microorganisms14051086

**Published:** 2026-05-11

**Authors:** Malihe Mehdizadeh Allaf, Tianxing Yi, Junhui Zhang, Shouyu Zhang, Kevin J. Erratt, Parham Dehnavi, Hassan Peerhossaini

**Affiliations:** 1 Department of Chemical and Biochemical Engineering, Thompson Engineering Building, Western University, 1151 Richmond Street N., London, ON N6A5B9, Canada; tyi7@uwo.ca (T.Y.); szha466@uwo.ca (S.Z.); 2 Department of Civil and Environmental Engineering, Western University, Spencer Engineering Building, 1151 Richmond Street N., London, ON N6A5B9, Canada; jzha2689@uwo.ca (J.Z.); pdehnavi@uwo.ca (P.D.); hpeerhos@uwo.ca (H.P.); 3 Department of Physical & Environmental Sciences, University of Toronto, Toronto, ON M5S 1A1, Canada; k.erratt@utoronto.ca; 4 Department of Mechanical & Materials Engineering, Western University, Spencer Engineering Building, 1151 Richmond Street N., London, ON N6A5B9, Canada; 5 Matière et Systèmes Complexes Laboratory, Université Paris Cité, CNRS UMR 7057, 75013 Paris, France

**Keywords:** algaecides, aquaculture, harmful algal blooms, plankton, management, hydrogen peroxide, copper sulfate, motility, mean squared displacement (MSD), probability density function (PDF)

## Abstract

Aquaculture is the fastest-growing food production sector, supplying more than half of the world’s seafood and projected to expand further to meet rising global protein demands. Among the various pressures confronting this industry, harmful algal blooms (HABs) rank among the most alarming. Ichthyotoxic flagellates are microalgae known for producing toxins or inducing gill damage that leads to widespread fish mortality. Their increasing frequency poses a critical threat to aquaculture, emphasizing the urgent need for effective and environmentally sustainable strategies to regulate and mitigate these harmful episodes. This study investigated the responses of three ichthyotoxic flagellates renowned for impacting aquaculture operations (*Prymnesium parvum*, *Heterosigma akashiwo*, and *Fibrocapsa japonica*) and tested their susceptibility to two routinely applied chemical agents, hydrogen peroxide (H_2_O_2_) and copper sulfate (CuSO_4_). Mortality, viability, and motility were assessed alongside trajectory-based metrics, including mean squared displacement (MSD) and probability density function (PDF). The results revealed species-specific sensitivities: *P. parvum* was highly susceptible to H_2_O_2_, while *H. akashiwo* and *F. japonica* were more susceptible to copper toxicity. Ichthyotoxic flagellates exhibited differential sensitivities to chemical treatments, with copper sulfate generally achieving lower EC_50_ thresholds and greater inhibition of motility than hydrogen peroxide, except in *P. parvum*. The rapid attenuation of motility at sublethal concentrations highlights swimming behavior as a functional vulnerability, reinforcing the potential for behavior-based mitigation strategies that minimize chemical loading and reduce unintended impacts on cultured fish and surrounding ecosystems.

## 1. Introduction

Aquaculture, the fastest-growing food production sector globally, now supplies over half of all seafood consumed globally, and output is expected to keep rising to meet the protein demands of a growing population [1,2]. The continued growth of this food sector is threatened by numerous environmental stressors, with harmful algal blooms (HABs) representing some of the most acute and costly challenges [3,4]. HABs are proliferations of phytoplankton species capable of producing toxins or causing other deleterious effects on aquatic ecosystems [5], and in the aquaculture context, they can result in extensive fish and shellfish mortality through physiological stress (e.g., gill damage, hemolytic compounds, and biotoxins) as well as indirect effects such as oxygen depletion following bloom senescence [6,7]. The economic ramifications of HABs in the aquaculture sector are substantial. Global HAB-related losses to aquaculture have been estimated at over USD 8 billion annually, with individual events costing tens to hundreds of millions of dollars in fish mortality and mitigative measures [4,8]. The combined influence of human activities and climate change is amplifying the threat posed by HABs, with evidence of rising frequency, broader geographic distribution, and heightened severity in many aquaculture-intensive regions. These trends necessitate urgent, evidence-based interventions to ensure the resilience of critical aquatic food supplies [3,6,9].

Among harmful algal taxa, ichthyotoxic flagellates represent an understudied yet problematic subset, receiving less research attention than toxin-producing species associated with shellfish poisoning [10,11,12]. Unlike many non-motile HAB taxa, these flagellates possess the ability for directed movement, enabling them to actively seek favorable conditions, aggregate around aquaculture cages, and accumulate on fish gills where their effects are most severe [3]. This behavioral advantage, combined with their diverse modes of action, makes them particularly concerning. Raphidophytes (e.g., *Heterosigma*, *Chattonellam*, and *Fibrocapsa*) [13,14,15,16], Dinophytes (e.g., *Karenia*) [17], or Prymnesiophyte (e.g., *Prymnesium*) [18] are all capable of inducing large-scale fish mortality. Their impacts arise through multiple mechanisms, including the production of ichthyotoxins, the generation of reactive oxygen species, and direct physical damage to gill tissues [4].

An array of tactics is used to mitigate the impacts of blooms and reduce losses in aquaculture [4]. Physical approaches include aeration and destratification to raise dissolved oxygen and disrupt surface accumulations, bubble curtains and skimmers to exclude or concentrate biomass, clay flocculation to sink cells from the water column, and targeted filtration at intakes [19,20]. Biological approaches seek to shift community structure or inhibit target taxa using probiotics and algicidal bacteria, selective grazers or filter feeders, and nutrient management that limits bloom-promoting conditions [21]. Chemical controls are often favored in operational settings because they provide rapid, scalable interventions that can be deployed in ponds, raceways, and net-pens with minimal infrastructure. Their appeal lies in immediacy and dose control, although they require careful stewardship to avoid non-target effects and post-treatment rebounds [22].

Among the chemical algaecides available, copper sulfate and hydrogen peroxide are two of the most frequently applied agents due to their accessibility, cost-effectiveness, and broad-spectrum algicidal activity [23]. Copper sulfate functions primarily through the release of bioavailable copper ions, which disrupt photosynthesis, enzyme function, and membrane integrity in phytoplankton cells. However, a critical concern associated with this agent is its potential to persist and accumulate in the environment, thereby increasing risks to other aquatic organisms [20,24]. In contrast, hydrogen peroxide acts by generating reactive oxygen species that cause oxidative damage to cell membranes, pigments, and organelles [25,26]. Hydrogen peroxide has attracted growing interest because of its rapid degradation in aquatic environments, which minimizes long-term environmental residues and reduces impacts on non-target organisms [27,28]. Nevertheless, the efficacy and success of hydrogen peroxide treatments are highly variable in scientific literature, likely influenced by the taxonomic composition of the bloom and environmental conditions such as temperature, salinity, and light availability [23,29,30].

There is a clear global research gap in understanding the differential responses of ichthyotoxic flagellates to treatments and the environmental conditions that regulate these responses and treatment outcomes. The aim of this study was twofold: (1) Determine taxonomic responses; ichthyotoxic flagellates have unique physiologies attributed to their diverse evolutionary lineages, making it necessary to identify which genera are most susceptible to these compounds in order to develop species-specific treatment regimens; and (2) characterize motility and behavioral dynamics under algaecide exposure; quantifying changes in swimming speed provides critical insight into the mechanistic effects of treatment at the cellular level. This helps explain whether impaired motility contributes to mortality and supports better predictions of treatment efficacy. In this study, we focus on three ichthyotoxic flagellates (*Prymnesium parvum*, *Heterosigma akashiwo*, and *Fibrocapsa japonica*) and assess their susceptibility to two commonly used chemical agents. The growth and motility metrics were assessed as indicators of physiological stress responses and determined the impacts on cell fitness and the median effective concentration (EC_50_).

## 2. Materials and Methods

### 2.1. Culturing and Experimental Procedures

Algal strains (Table 1) were obtained from the Algal Resources Collection (ARC), Wilmington, NC, USA. Stock cultures were maintained in 125 mL Erlenmeyer flasks, supplied with a chemically defined medium (Table 1) prepared according to [31]. Cultures were incubated at 20 ± 1 °C under a 12:12 h light–dark photoperiod with an irradiance of 75 ± 5 μmol photons m^−2^ s^−1^.

Analytical-grade hydrogen peroxide (30% H_2_O_2_; Fisher Chemical, Pittsburgh, PA, USA) and copper sulfate (CuSO_4_·5H_2_O; Sigma-Aldrich, St. Louis, MO, USA) were used to prepare stock solutions immediately prior to each experiment. Independent exposure experiments were conducted for H_2_O_2_ and CuSO_4_·5H_2_O. These stock solutions were added to 30 mL aliquots of mid-exponential phase algal cells (initial cell density: 1 × 10^4^ cells mL^−1^) to achieve final concentrations of 0, 0.625, 1.25, 2.5, 5, 10, and 20 mg L^−1^ for both H_2_O_2_ and CuSO_4_·5H_2_O. Each concentration was tested in triplicate, and algal bioassay conditions for light and temperature were consistent with the culturing procedures described above.

### 2.2. Cellular Mortality

Cell mortality was evaluated after 24 h via flow cytometric analysis, Cytek^®^ Guava^®^ Muse^®^ Cell Analyzer (Fremont, CA, USA). A sub-sample (100 μL) of each culture was mixed with 400 μL of Muse^®^ Count & Viability Reagent and incubated at room temperature for 5 min, and then analyzed by the Cell Analyzer. Cell populations were identified and gated using a dot-plot, and cell mortality was calculated according to the following formula:

(1) $$Cell\;Mortality\;\left(\%\right)=\frac{Dead\;cells}{Total\;cells}\times 100$$

The half-maximal effective concentration (EC_50_) for each compound was derived from variable-slope sigmoidal concentration–response analysis. Curve fitting and parameter estimation were conducted using OriginPro 2017 (OriginLab, Northampton, MA, USA).

### 2.3. Cellular Motility

A 20 μL aliquot of each sample was placed on a concave glass slide and covered with a coverslip. Imaging was performed using a Nikon Eclipse Ti2 inverted microscope equipped with a 10× objective lens and an Iris 15 monochrome camera at 16-bit depth and a spatial resolution of 1.7 μm pixel^−1^. Recordings were acquired for 60 s at 30 frames s^−1^ with a frame resolution of 1264 × 740 pixels and stored in .mp4 format. To facilitate motion analysis, raw video recordings were processed to generate 15 s clips with enhanced contrast. Processed video recordings were then analyzed using TrackPy (version 0.7), a Python (version 3.12)-based particle tracking toolkit. Frame-by-frame analysis identified individual cell positions, and trajectories were reconstructed by linking positions across consecutive frames.

In addition, cell dynamics were quantified by calculating the mean squared displacement (MSD) and displacement probability density function (PDF). The MSD characterizes how far a cell moves over time, providing information on both the magnitude and temporal features of its motion [32]. The PDF describes the distribution of displacement steps over a defined time interval, offering insight into the variability and typical patterns of cell movement, including the presence of directed or irregular motility [32]. Together, these metrics allowed a detailed comparison of the dynamic behavior across species. Each sample was analyzed in quintuplicate.

### 2.4. Statistical Analysis

Normality and sphericity were assessed using the Shapiro–Wilk and Mauchly’s tests, respectively. Significant differences among treatment means were evaluated using repeated measures Analysis of Variance (ANOVA), followed by Tukey’s post hoc test, implemented in OriginPro 2017 (OriginLab, USA). Statistical significance was determined at *p*-value < 0.05.

## 3. Results and Discussion

The increasing occurrence of phytoflagellate species associated with ichthyotoxic events has intensified the demand for effective mitigation approaches within aquaculture systems. Among available strategies, copper sulfate and hydrogen peroxide remain widely used due to their rapid and cost-efficient action, yet they exert their effects through distinct metal-induced and oxidative mechanisms that differentially impair algal physiology, including cellular integrity and motility [20,24,25,26]. Given the central role of motility in phytoflagellate ecology, influencing resource acquisition, predator avoidance, and bloom development, evaluating motility responses to algicide exposure provides an opportunity to detect sublethal behavioral shifts that may contribute to bloom suppression. Such motility-based indicators can serve as early-warning metrics, offering a practical tool for assessing the subtle, pre-lethal effects of algicides in bloom-management strategies. Understanding how these two algicides influence phytoflagellate behavior is therefore critical for assessing their ecological impact and informing more sustainable management practices.

### 3.1. Cellular Mortality Assessment

**Figure 1:**
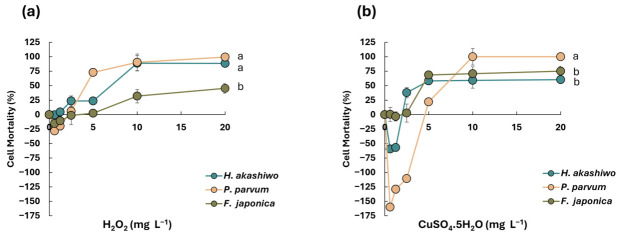
Cell mortality after 24 h of exposure to various concentrations of hydrogen peroxide (**a**) and copper sulfate (**b**). The presented data for each species is the average of different datasets ± SD. Different lowercase letters indicate statistically significant differences among species at the highest tested concentration (20 mg L^−1^). The same lowercase letter denotes no significant difference. Statistical significance was determined at a *p*-value < 0.05 level.

Hydrogen peroxide induced a clear concentration-dependent increase in cell mortality across all three HABs species examined (Figure 1a). *P. parvum* exhibited the highest sensitivity, with mortality approaching 100% at concentrations of approximately 10 mg L^−1^. *H. akashiwo* showed a moderate increase in mortality, while *F. japonica* displayed a biphasic response, characterized by an initial decline or apparent growth at lower concentrations, followed by a gradual rise in mortality at higher concentrations. Statistical analysis revealed a significant main effect of algal species on mortality responses (*p*-value < 0.05). Post hoc pairwise comparisons indicated that both *H. akashiwo* and *P. parvum* exhibited significantly higher mortality than *F. japonica* (*p*-value < 0.05), whereas no significant difference was observed between *H. akashiwo* and *P. parvum*. These results indicate that *F. japonica* is less sensitive to the applied oxidative stress than the other two species, suggesting species-specific physiological or structural mechanisms underlying their sensitivity to oxidative stress.

In contrast, copper sulfate treatment caused more variable responses (Figure 1b). *P. parvum* and *H. akashiwo* demonstrated a decline in mortality at low concentrations, reaching negative values suggestive of potential growth stimulation, physiological adaptation, or experimental variability, before stabilizing near zero at higher concentrations. *F. japonica* responded more predictably, with mortality increasing steadily and plateauing at elevated CuSO_4_ concentrations, indicating a saturation effect.

These results highlight species-specific sensitivities to chemical treatments. H_2_O_2_ was particularly effective against *P. parvum*, while CuSO_4_ showed greater efficacy at lower concentrations against *F. japonica* and *H. akashiwo*. Notably, concentrations exceeding 10 mg L^−1^ produced strong effects on *P. parvum* under both treatments, suggesting a potential threshold for effective control. Consistent with the two-way repeated-measures ANOVA and subsequent post hoc analysis, the mortality rate of *P. parvum* was significantly higher than both *H. akashiwo* and *F. japonica*, with *p*-values of 0.005 and 0.001, respectively.

The differential responses observed among various species under different treatments reflect not only species-specific biochemical sensitivity but also underlying morphological traits that influence chemical interactions and stress tolerance. *P. parvum*, a haptophyte with a smooth ellipsoid cell surface and a haptonema, is covered only by thin organic scales that offer minimal structural defense, leaving its plasma membrane directly exposed to oxidative agents [33,34]. This structural vulnerability, combined with its high surface-area-to-volume ratio and motile appendages, likely contributes to its pronounced sensitivity to hydrogen peroxide and copper ions [33,34]. In contrast, *H. akashiwo* lacks a rigid wall but contains mucocysts beneath the plasma membrane, which may serve as a protective barrier, delaying oxidative damage and potentially chelating copper ions before they reach vital cellular components [35]. *F. japonica*, though lacking a true wall, is distinguished by fibrous extracellular material and mucocysts that may slow down the diffusion of hydrogen peroxide and intercept copper ions, explaining its delayed and biphasic response to chemical exposure [36].

### 3.2. Phytoplankton Toxicological Response to H_2_O_2_ and CuSO_4_

The EC_50_ widely acts as a robust parameter for evaluating the potency of chemical agents in suppressing biological activity, including phytoplankton growth. It serves as a critical threshold for determining the concentration at which a compound causes a 50% reduction in biomass, making it highly relevant for environmental risk assessments and mitigation strategies targeting HABs [23,37].

In this study, the EC_50_ values were calculated after 24 h of exposure to H_2_O_2_ and CuSO_4_ (Table 2). Among the tested species, *F. japonica* exhibited the highest tolerance under H_2_O_2_ treatment, followed by *H. akashiwo*, while *P. parvum* was the most sensitive species. Statistical analysis confirmed significant differences between *P. parvum* and the other two species (*p*-value < 0.05). Under CuSO_4_ treatment, the toxicity profile shifted. *P. parvum* showed increased tolerance, while *H.akashiwo* was the most sensitive species (*p*-value < 0.05).

These findings underscore the importance of incorporating psychological diversity into HAB management strategies. The differential sensitivity of phytoplankton taxa to chemical treatments such as H_2_O_2_ and CuSO_4_ indicates that mitigation approaches need to be tailored to bloom composition [23,37,38,39]. Raphidophyte taxa exhibited convergent response profiles, characterized by lower EC_50_ values and greater overall treatment sensitivity, whereas *P. parvum* (Haptophyta) displayed greater susceptibility to hydrogen peroxide. These findings demonstrate that, despite being broadly classified as ichthyotoxic flagellates, taxa from distinct phycological divisions can differ substantially in their physiological responses to chemical stressors. This divergence emphasizes the necessity of resolving bloom composition at finer taxonomic levels and tailoring mitigation strategies to species-specific sensitivities rather than relying on broad functional generalizations.

### 3.3. Monitoring Algal Motility Following Chemical Treatment

**Figure 2:**
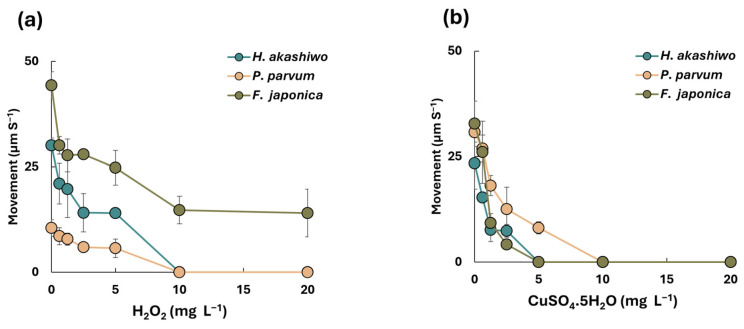
Cell movement after 24 h of exposure to various concentrations of hydrogen peroxide (**a**) and copper sulfate (**b**). The presented data for each species is the average of different datasets ± SD.

Due to the morphological structure of phytoflagellates, monitoring motility behavior under algaecide exposure is critical for understanding the mechanistic effect of treatment on phytoflagellates and predicting their ecological impacts. After 24 h of treatment, the average velocity of phytoplankton was measured and plotted against algaecide concentrations (Figure 2). It was observed that the motility of all three species showed a clear dose-dependent response to both H_2_O_2_ and CuSO_4_. Increasing concentrations of either algaecide resulted in significant reductions in motility (*p*-value < 0.01), with complete cessation of movement observed at the highest doses, except for F. *japonica* treated by H_2_O_2_.

Under H_2_O_2_ treatment, *P. parvum* exhibited the highest sensitivity (*p*-value < 0.01), followed by *H. akashiwo* and *F. japonica*. At 10 mg L^−1^, *F. japonica* retained approximately 50% of its initial motility, whereas *P. parvum* and *H. akashiwo* showed complete loss of movement. In contrast, under CuSO_4_ exposure, the trend shifted: *H. akashiwo* and *F. japonica* displayed similar reductions in motility, followed by *P. parvum* (*p*-value < 0.01). Both *H. akashiwo* and *F. japonica* stopped moving at 5 mg L^−1^, while *P. parvum* required 10 mg L^−1^ for complete immobilization.

The observed dose-dependent decline in motility across all species confirms that oxidative and ionic stressors disrupt key physiological processes required for flagellar function. Motility impairment is an early and sensitive indicator of cellular stress, preceding cell growth inhibition and death under oxidative treatments [40]. Hydrogen peroxide exerts its algacidal effect primarily through oxidative damage to membranes and the flagellar apparatus, generating ROS that impair ion gradients and ATP-dependent motility systems [41,42]. Copper ions, on the other hand, interfere with enzyme activity and cellular homeostasis, leading to rapid immobilization and eventual cell lysis [43,44,45].

Species-specific differences in sensitivity reflect underlying physiological and biochemical traits. Although *P. parvum* has a smaller size (≤10 µm) and biovolume (368 µm^3^) compared to *H. akashiwo* and *F. japonica*, its surface area-to-volume ratio is higher [46,47,48]. This morphological characteristic increases the relative exposure of *P. parvum* to oxidizing agents such as hydrogen peroxide, potentially accelerating oxidative stress and cellular damage. On the other hand, the similar response of *H. akashiwo* and *F. japonica* to CuSO_4_ suggests shared tolerance mechanisms, possibly linked to metal-binding proteins and detoxification pathways common in raphidophytes [46,49].

Importantly, motility collapse at higher doses indicates that behavioral endpoints can serve as rapid predictors of treatment efficacy. This supports the integration of real-time motility monitoring into HAB management strategies, enabling adaptive dosing before irreversible ecological impacts occur. Previous work has shown that motility-based thresholds correlate with mortality and can inform operational decisions in aquaculture systems [40,50].

### 3.4. Cell Motion Dynamics: Mean Squared Displacement (MSD) and Probability Density Function (PDF)

The mean squared displacement (MSD) is a quantitative measure of the average squared distance traveled by a particle over time [32], and is calculated using the following equation:

(2) $$MSD\;\left(t\right)=\left\langle {\left[X_{i}\left(t\right)-X_{i}\left(0\right)\right]}^{2}\right\rangle$$

where $X_{i}\left(t\right)$ denotes the positions of particle *i* at time and $\left\langle .\right\rangle$ denotes ensemble average.

**Figure 3:**
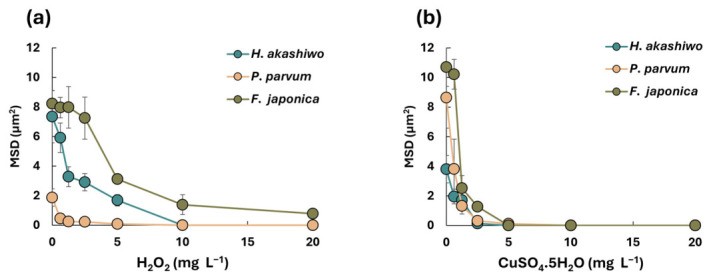
Variation in MSD across different species under varying concentrations of H_2_O_2_ (**a**) and CuSO_4_ (**b**) after 24 h. The presented data for each species is the average of different datasets ± SD.

Increasing the concentrations of H_2_O_2_ and CuSO_4_ resulted in a pronounced decline in MSD for all three species after 24 h (Figure 3a,b). In line with previous results, even low concentrations of CuSO_4_ significantly impaired motility and therefore MSD in all species (*p*-value < 0.01).

**Figure 4:**
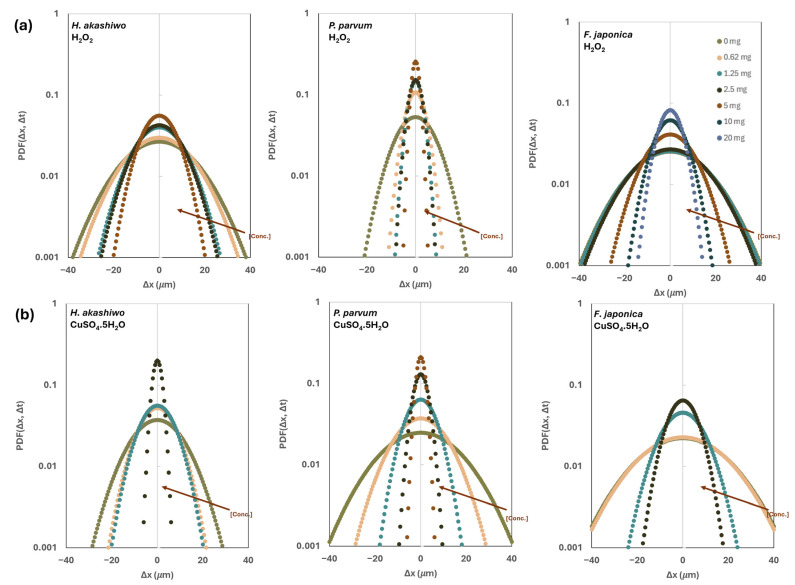
Displacement probability density function (PDF) of cell displacement under increasing concentrations of H_2_O_2_ (**a**) and CuSO_4 _(**b**). Over 24 h. Narrower curves at higher concentrations suggest reduced motility, while broader curves at lower concentrations indicate more active movement. The arrow indicates the direction of increasing algaecide concentration [Conc.]. The presented data for each species is the average of different datasets ± SD.

The probability density function (PDF) analysis of the movement also confirmed a clear concentration-dependent effect of both algaecides on the motility behavior of the studied phytoflagellates (Figure 4). Across all treatments, progressive narrowing of PDFs indicated reduced displacement and motility, with the degree of collapse varying by species and chemical stressors.

Under H_2_O_2_ exposure, *P. parvum* exhibited the most pronounced motility reduction, with PDFs sharply contracting even at low concentrations (0.625–2.5 mg L^−1^), confirming its high susceptibility to oxidative stress. Meanwhile, *H. akashiwo* and *F. japonica* maintained broader displacement distributions at lower doses but showed severe narrowing at 10–20 mg L^−1^, signaling progressive impairment.

Under CuSO_4_ treatment, *H. akashiwo* and *P. parvum* displayed marked motility collapse at intermediate concentrations (5–10 mg L^−1^), while *F. japonica* retained relatively broader PDFs across similar doses.

PDF analysis complements MSD by revealing the distributional characteristics of cell movement rather than just average displacement. The observed narrowing of PDFs under stress indicates a shift from active swimming to constrained or erratic motion, consistent with stress-induced impairment of flagellar activity and energy metabolism [51,52].

Overall, these results confirm that both algaecides significantly impair motility in the studied ichthyotoxic flagellates, but the magnitude and rate of inhibition differ between stressors and species. CuSO_4_ exhibited a more potent and rapid effect, likely due to its ability to disrupt cellular ion homeostasis and enzyme function, leading to immediate physiological stress [24,53,54]. In contrast, H_2_O_2_-induced oxidative damage [25,26] appears to accumulate over time, causing a more gradual decline in motility.

## 4. Conclusions

This study demonstrated distinct, species-specific sensitivities of each ichthyotoxic flagellate to commonly used chemical mitigation agents. Our findings highlight that all species exhibited sensitivity to both algaecides, but with distinct patterns: *P. parvum* was highly vulnerable to oxidative stress, likely due to its high surface-area-to-volume ratio and limited structural defenses, whereas *H. akashiwo* and *F. japonica* showed greater tolerance to H_2_O_2_ but increased susceptibility to copper toxicity, reflecting shared detoxification mechanisms among raphidophytes.

The findings of this study suggest that H_2_O_2_ may represent a more suitable mitigation option for aquaculture settings, particularly when used at low to moderate concentrations and short exposure durations. Hydrogen peroxide offers a more environmentally responsible alternative to copper sulfate, largely because it degrades rapidly and poses minimal risk of sediment accumulation [55]. In contrast, although copper sulfate is effective, its continued use raises concerns regarding sediment buildup, toxicity to non-target organisms, and increasing regulatory constraints [53].

Importantly, when these treatments are evaluated through the lens of motility-based mechanisms, the ability of flagellated taxa to swim and actively position themselves within the water column becomes a critical determinant of both their vulnerability to intervention and the development of targeted, ecologically responsible control strategies. The findings of this study suggest that algal motility can be disrupted at lower concentrations than those needed to induce significant cell mortality, pointing to an underexplored strategy for mitigating harmful algal blooms.

Rather than relying on high-dose treatments aimed at cell death, disrupting swimming behavior could limit the ability of these taxa to position themselves in harmful zones (e.g., aggregate around cages). By removing their capacity to navigate the water column, we effectively neutralize their ecological advantage and diminish bloom impact without the need for more aggressive interventions. It should also be noted that all experiments in this study were conducted under controlled laboratory conditions. Accordingly, future studies should evaluate these responses under more complex and representative natural environmental conditions to assess their relevance beyond laboratory-scale systems.

## Figures and Tables

**Table 1:** Experimental algal strains and their proper culturing media.

Algae Species	Culture Strain Number	Media
*Prymnesium parvum*	ARC 66	f/20
*Heterosigma akashiwo*	ARC 446	f/2
*Fibrocapsa japonica*	ARC 40	f/2

**Table 2:** The median effective concentration (EC_50_) values (mg L^−1^) for different species after 24 h exposure to H_2_O_2_ and CuSO_4_.

Time (h)	Phytoplankton	Hydrogen Peroxide (EC_50_)	Copper Sulfate (EC50)
24	*H. akashiwo*	6.01 ± 1.51	2.05 ± 0.44
	*P. parvum*	3.35 ± 0.17	4.07 ± 0.4
	*F. japonica*	7.86 ± 2.94	3.55 ± 0.42

## Data Availability

The original contributions presented in this study are included in the article. Further inquiries can be directed to the corresponding author.

## Abbreviations

The following abbreviations are used in this manuscript:

HABs	Harmful algal blooms
H_2_O_2_	Hydrogen peroxide
CuSO_4_	Copper sulfate
MSD	Mean squared displacement
PDF	Probability density function
EC_50_	Median effective concentration
ANOVA	Analysis of variance

## References

[1] Subasinghe R., Soto D., Jia J. (2009). Global aquaculture and its role in sustainable development. Rev. Aquac..

[2] Naylor R.L., Hardy R.W., Buschmann A.H., Bush S.R., Cao L., Klinger D.H., Little D.C., Lubchenco J., Shumway S.E., Troell M. (2021). A 20-year retrospective review of global aquaculture. Nature.

[3] Brown A.R., Lilley M., Shutler J., Lowe C., Artioli Y., Torres R., Berdalet E., Tyler C.R. (2020). Assessing risks and mitigating impacts of harmful algal blooms on mariculture and marine fisheries. Rev. Aquac..

[4] Mehdizadeh Allaf M., Erratt K.J. (2024). Navigating aquaculture losses: Tackling fish-killing phytoflagellates in a changing global landscape. Rev. Aquac..

[5] Smayda T.J. (1997). What is a bloom? A commentary. Limnol. Oceanogr..

[6] Hallegraeff G.M., Hallegraeff G.M., Anderson D.M., Cembella A.D. (2004). Harmful algal blooms: A global overview. Manual on Harmful Marine Microalgae.

[7] Fernandes-Salvador J.A., Davidson K., Sourisseau M., Revilla M., Schmidt W., Clarke D., Miller P.I., Arce P., Fernández R., Maman L. (2021). Current status of forecasting toxic harmful algae for the north-east Atlantic shellfish aquaculture industry. Front. Mar. Sci..

[8] Díaz P.A., Álvarez G., Varela D., Pérez-Santos I., Díaz M., Molinet C., Seguel M., Aguilera-Belmonte A., Guzmán L., Uribe E. (2019). Impacts of harmful algal blooms on the aquaculture industry: Chile as a case study. Perspect. Phycol..

[9] Mehdizadeh Allaf M., Trick C.G. (2025). Growth response and cell permeability of the fish-killing phytoflagellate *Heterosigma akashiwo* under projected climate conditions. Toxins.

[10] Mehdizadeh Allaf M. (2023). *Heterosigma akashiwo*, a fish-killing flagellate. Microbiol. Res..

[11] Whyte J.N.C., Haigh N., Ginther N.G., Keddy L.J. (2001). First record of blooms of Cochlodinium sp. (Gymnodiniales, Dinophyceae) causing mortality to aquacultured salmon on the west coast of Canada. Phycologia.

[12] Davidson K., Gowen R.J., Harrison P.J., Fleming L.E., Hoagland P., Moschonas G. (2014). Anthropogenic nutrients and harmful algae in coastal waters. J. Environ. Manag..

[13] Chang F.H., Anderson C., Boustead N.C. (1990). First record of a *Heterosigma* (raphidophyceae) bloom with associated mortality of cage-reared salmon in big glory bay, New zealand. New Zeal. J. Mar. Freshw. Res..

[14] García-Mendoza E., Cáceres-Martínez J., Rivas D., Fimbres-Martinez M., Sánchez-Bravo Y., Vásquez-Yeomans R., Medina-Elizalde J. (2018). Mass Mortality of Cultivated Northern Bluefin Tuna Thunnus *thynnus orientalis* associated with *Chattonella* Species in Baja California, Mexico. Front. Mar. Sci..

[15] Jack Rensel J.E., Haigh N., Tynan T.J. (2010). Fraser river sockeye salmon marine survival decline and harmful blooms of *Heterosigma akashiwo*. Harmful Algae.

[16] Pezzolesi L., Cucchiari E., Guerrini F., Pasteris A., Galletti P., Tagliavini E., Totti C., Pistocchi R. (2010). Toxicity evaluation of *Fibrocapsa japonica* from the Northern Adriatic Sea through a chemical and toxicological approach. Harmful Algae.

[17] Li X., Yan T., Yu R., Zhou M. (2019). A review of *Karenia mikimotoi*: Bloom events, physiology, toxicity and toxic mechanism. Harmful Algae.

[18] Sobieraj J., Metelski D. (2023). Insights into toxic *Prymnesium parvum* blooms as a cause of the ecological disaster on the Odra river. Toxins.

[19] Anderson D.M. (2009). Approaches to monitoring, control and management of harmful algal blooms (HABs). Ocean Coast. Manag..

[20] Jančula D., MarŠálek B. (2011). Critical review of actually available chemical compounds for prevention and management of cyanobacterial blooms. Chemosphere.

[21] Balaji-Prasath B., Wang Y., Su Y.P., Hamilton D.P., Lin H., Zheng L., Zhang Y. (2022). Methods to control harmful algal blooms: A review. Environ. Chem. Lett..

[22] Gallardo-Rodríguez J.J., Astuya-Villalón A., Llanos-Rivera A., Avello-Fontalba V., Ulloa-Jofré V. (2019). A critical review on control methods for harmful algal blooms. Rev. Aquac..

[23] Mehdizadeh Allaf M., Erratt K.J., Peerhossaini H. (2023). Comparative assessment of algaecide performance on freshwater phytoplankton: Understanding differential sensitivities to frame cyanobacteria management. Water Res..

[24] Ebenezer V., Lim W.A., Ki J.S. (2014). Effects of the algicides CuSO_4_ and NaOCl on various physiological parameters in the harmful dinoflagellate *Cochlodinium polykrikoides*. J. Appl. Phycol..

[25] Burson A., Matthijs H.C.P., de Bruijne W., Talens R., Hoogenboom R., Gerssen A., Visser P.M., Stomp M., Steur K., van Scheppingen Y. (2014). Termination of a toxic Alexandrium bloom with hydrogen peroxide. Harmful Algae.

[26] Chen Y., Zaman F., Jia Y., Huang Y., Li T., Bai F., Li L., Song L., Li J. (2024). Harmful cyanobacterial bloom control with hydrogen peroxide: Mechanism, affecting factors, development, and prospects. Curr. Pollut. Rep..

[27] Drábková M., Admiraal W., Maršálek B. (2007). Combined exposure to hydrogen peroxide and light-selective effects on cyanobacteria, green algae, and diatoms. Environ. Sci. Technol..

[28] Drábková M., Matthijs H.C.P., Admiraal W., Maršálek B. (2007). Selective effects of H_2_O_2_ on cyanobacterial photosynthesis. Photosynthetica.

[29] Sandrini G., Piel T., Xu T., White E., Qin H., Slot P.C., Huisman J., Visser P.M. (2020). Sensitivity to hydrogen peroxide of the bloom-forming cyanobacterium *Microcystis* PCC 7806 depends on nutrient availability. Harmful Algae.

[30] Buley R.P., Gladfelter M.F., Fernandez-Figueroa E.G., Wilson A.E. (2023). Complex effects of dissolved organic matter, temperature, and initial bloom density on the efficacy of hydrogen peroxide to control cyanobacteria. Environ. Sci. Pollut. Res..

[31] Andersen R.A., Berges J.A., Harrison P.J., Watanabe M.M., Andersen R.A. (2005). Recipes for freshwater and seawater media. Algal Culturing Techniques.

[32] Samadi Z., Mehdizadeh Allaf M., Vourc’h T., DeGroot C.T., Peerhossaini H. (2023). Investigation of *Synechocystis* sp. CPCC 534 motility during different stages of the growth period in active fluids. Processes.

[33] Roelke D.L., Barkoh A., Brooks B.W., Grover J.P., Hambright K.D., Laclaire J.W., Moeller P.D.R., Patino R. (2016). A chronicle of a killer alga in the west: Ecology, assessment, and management of *Prymnesium parvum* blooms. Hydrobiologia.

[34] Wagstaff B.A., Hems E.S., Rejzek M., Pratscher J., Brooks E., Kuhaudomlarp S., O’Neill E.C., Donaldson M.I., Lane S., Currie J. (2018). Insights into toxic *prymnesium parvum* blooms: The role of sugars and algal viruses. Biochem. Soc. Trans..

[35] Engesmo A., Eikrem W., Seoane S., Smith K., Edvardsen B., Hofgaard A., Tomas C.R. (2016). New insights into the morphology and phylogeny of *Heterosigma akashiwo* (Raphidophyceae), with the description of *Heterosigma minor* sp. nov. Phycologia.

[36] de Boer M.K., Tyl M.R., Fu M., Kulk G., Liebezeit G., Tomas C.R., Lenzi A., Naar J., Vrieling E.G., van Rijssel M. (2009). Haemolytic activity within the species Fibrocapsa japonica (Raphidophyceae). Harmful Algae.

[37] Wang H., Ebenezer V., Ki J.S. (2018). Photosynthetic and biochemical responses of the freshwater green algae *Closterium ehrenbergii Meneghini* (Conjugatophyceae) exposed to the metal coppers and its implication for toxicity testing. J. Microbiol..

[38] Hu J., Berthold D.E., Wang Y., Xiao X., Laughinghouse H.D. (2022). Treatment of the red tide dinoflagellate *Karenia brevis* and brevetoxins using USEPA-registered algaecides. Harmful Algae.

[39] Lusty M.W., Gobler C.J. (2020). The Efficacy of Hydrogen Peroxide in Mitigating Communities across Four Lakes in NY, USA. Toxins.

[40] Park S.C., Lee J.K., Kim S.W., Park Y. (2011). Selective algicidal action of peptides against harmful algal bloom species. PLoS ONE.

[41] Wagstaff B.A., Pratscher J., Rivera P.P.L., Hems E.S., Brooks E., Rejzek M., Todd J.D., Murrell J.C., Field R.A. (2021). Assessing the toxicity and mitigating the impact of harmful *Prymnesium* blooms in eutrophic waters of the Norfolk broads. Environ. Sci. Technol..

[42] Kim C.S., Lee S.G., Lee C.K., Kim H.G., Jung J. (1999). Reactive oxygen species as causative agents in the ichthyotoxicity of the red tide dinoflagellate *Cochlodinium polykrikoides*. J. Plankton Res..

[43] Liu G., Chai X., Shao Y., Hu L., Xie Q., Wu H. (2011). Toxicity of copper, lead, and cadmium on the motility of two marine microalgae *Isochrysis galbana* and *Tetraselmis chui*. J. Environ. Sci..

[44] Tsai K.P. (2016). Management of target algae by using copper-based algaecides: Effects of algal cell density and sensitivity to copper. Water Air Soil Pollut..

[45] Qian H., Yu S., Sun Z., Xie X., Liu W., Fu Z. (2010). Effects of copper sulfate, hydrogen peroxide and N-phenyl-2-naphthylamine on oxidative stress and the expression of genes involved photosynthesis and microcystin disposition in *Microcystis aeruginosa*. Aquat. Toxicol..

[46] Moreno-Andrés J., Romero-Martínez L., Seoane S., Acevedo-Merino A., Moreno-Garrido I., Nebot E. (2023). Evaluation of algaecide effectiveness of five different oxidants applied on harmful phytoplankton. J. Hazard. Mater..

[47] Olenina I., Hajdu S., Edler L., Andersson A., Wasmund N., Busch S., Göbel J., Gromisz S., Huseby S., Huttunen M. (2006). Biovolumes and size-classes of phytoplankton in the Baltic Sea. Proceedings of the HELCOM Baltic Sea Environment Proceedings.

[48] Aguiar Juárez D., Sunesen I., Flores-Leñero A., Norambuena L., Krock B., Fuenzalida G., Mardones J.I. (2025). Uncovering *Fibrocapsa japonica* (Raphidophyceae) in South America: First taxonomic and toxicological Insights from Argentinean coastal waters. Toxins.

[49] Markina Z.V. (2021). The cell ultrastructure and autotrophic function of the Raphidophyte alga *Heterosigma akashiwo* (Y. Hada) Y. Hada ex Y. Hara and M. Chihara, 1987 under copper exposure. Russ. J. Mar. Biol..

[50] Du X., Zhou W., Zhang W., Sun S., Han Y., Tang Y., Shi W., Liu G. (2021). Toxicities of three metal oxide nanoparticles to a marine microalga: Impacts on the motility and potential affecting mechanisms. Environ. Pollut..

[51] Giugliano G., Valentino M., Cavalletti E., Memmolo P., Miccio L., Bianco V., Sardo A., Ferraro P. (2025). Digital holography unveils sub-lethal copper doses using motility patterns of *Tetraselmis* microalgae bioprobes. Algal Res..

[52] Bondoc-Naumovitz K.G., Laeverenz-Schlogelhofer H., Poon R.N., Boggon A.K., Bentley S.A., Cortese D., Wan K.Y. (2023). Methods and measures for investigating microscale motility. Proceedings of the Integrative and Comparative Biology.

[53] Leal P.R., Moschini-Carlos V., López-Doval J.C., Cintra J.P., Yamamoto J.K., Bitencourt M.D., Santos R.F., Abreu G.C., Pompêo M.L.M. (2018). Impact of copper sulfate application at an urban Brazilian reservoir: A geostatistical and ecotoxicological approach. Sci. Total Environ..

[54] Tsai K.P., Uzun H., Chen H., Karanfil T., Chow A.T. (2019). Control wildfire-induced *Microcystis aeruginosa* blooms by copper sulfate: Trade-offs between reducing algal organic matter and promoting disinfection byproduct formation. Water Res..

[55] Matthijs H.C.P., Visser P.M., Reeze B., Meeuse J., Slot P.C., Wijn G., Talens R., Huisman J. (2012). Selective suppression of harmful cyanobacteria in an entire lake with hydrogen peroxide. Water Res..

